# Three Memoirs on the Development and Structure of the Teeth and Epithelium, Read at the Ninth Annual Meeting of the British Association in August 1839; with the Diagrams Exhibited in Illustration of Them

**Published:** 1841-10

**Authors:** 


### ART. XIII.

*Three Memoirs on the Development and Structure of the Teeth and Epithelium, read at the Ninth Annual Meeting of the British Association in August* 1839; *with the Diagrams exhibited in illustration of them.* By ALEXANDER NASMYTH, F.L.S. F.G.S. &c.—*London,* 1841. 8vo, pp. 47.

IT appears, from a long but temperately-written introduction to this small work, that the abstract of the memoirs of which it consists, after having been printed with a view to publication in the Transactions of the Association, was afterwards suppressed, on what certainly appears to us most insufficient grounds, and, notwithstanding the author's remonstrances, has not yet been published by the Association. We have neither time nor inclination to enter into the merits of the controversy between the author and the council of the Association, though we think it clear that he has been very ill treated. Our space will permit us merely to give a condensed analysis of the contents of the memoirs, which we submit to our readers without other remark than that Mr. Nasmyth deserves the highest credit for the zeal, industry, and intelligence with which he appears to have conducted his researches.

*The Pulp.* This is principally composed of cells or vesicles, varying extremely in size and shape, and disposed in layers. Its substance is traversed by vessels of which the direction is generally vertical. The vessels do not penetrate the reticular surface of the pulp, but are still so closely connected with it that they are often lacerated in attempting to separate the ivory from the pulp. The nature of the contents of the cells cannot be exactly ascertained. They frequently shrink or collapse to such an extent that the pulp nearly disappears, and this seems to take place more decidedly when the tooth has been in a healthy state, and more frequently in adult than in temporary teeth. In the body of the pulp they are arranged in ill-defined layers; at its surface they are more regularly disposed, more distinctly cellular, and are arranged in reticular leaflets; in short, at the surface they are prepared by some peculiar change in their form and arrangement for the reception of earthy matter, or in other words for their transition into ivory. The compartments of this beautiful reticular, formative surface are oval and overlap one another. Each compartment on being insulated is found to be curiously and regularly organized. This reticular conformation of the surface of the pulp presents peculiar diversities in different animals. Mr. Nasmyth first observed them in the human subject; afterwards in all other animals which he examined, varying in size and arrangement in different cases. In the human subject the reticular leaflets have a well-defined scolloped border, on which at regular intervals processes are observed. It is into the cells of these reticular leaflets that the osseous matter is deposited, constituting the ivory of the tooth; the precise mode in which this process is effected Mr. Nasmyth has not been able to discover. On the completion of this process the leaflet becomes a layer of perfect ivory, in connexion with the subjacent reticular leaflet, which is now in its turn become part of the formative surface of the pulp; by the time that this latter has been ossified, the same process has commenced in the leaflet beneath it; and the same phenomena are renewed in each successive leaflet or layer until the whole of the tooth is formed. Mr. Nasmyth's diagrams in illustration of this interesting portion of his subject are *fac-similes* of preparations showing the surface of the pulp under a variety of circumstances. In some it is entire; in others it is more or less damaged by disruption from the already ossified layers above, and is strewed with cellular fragments of those layers, and presents also patches denoting extravasated fluid containing probably osseous matter. The whole appearances are those of a peculiar system of cells external to the peripheral ramifications of the blood-vessels, the functions of which are highly interesting, and the study of which will ultimately lead, the author thinks, to the unveiling of many vital processes connected with the growth of animal tissues, which are at present shrouded in obscurity.

The cells of the reticular surface appear, during their transformation into ivory, to be subdivided into minute cellules, for they present the appearance of being filled with granules in progressive stages of development. The reticular cells, as is shown in Mr. Nasmyth's diagrams, have a regular fibrous framework, which, he thinks, becomes, on the deposition of ossific matter within the former, the fibres of the ivory. The diameter of the fibrous framework is precisely that of the fibres of the ivory, and the points or projections observed on the surface of the pulp, when the superincumbent ivory has been separated from it, may be traced to belong to the substance of the latter. Whilst forming a portion of the pulp these fibres are in a great measure coiled up; on the distension of the interfibrous cells by ossific matter they are drawn out, but still continue to a certain extent spirally curved.

The foregoing may be regarded as the synthetic portion of the author's observations; we now proceed to his analytic researches; for not contented with tracing the formation of ivory by the ossific transition of the pulp, he has followed out his investigations by restoring it as far as possible to what may be termed its pulpal state, by submitting it to the action of acid, and thus emptying the cells of their ossific contents. One of his diagrams shows the appearance of the ivory when the earthy matter has been almost entirely removed by acid, but where the cells still retain their position, general appearance, and connexion with each other. Another represents a more advanced stage of decomposition where there seem to be attached to each fibre minute lateral filaments, which he presumes to be the remaining portions of the emptied cells. He compares these cells in the former diagram, where they retain the erect distended form which they acquired by the deposition within them of ossific matter, with their original state in the reticular surface of the pulp before its ossification, where they lie collapsed one above another. The fibres of the ivory, on being submitted to the action of acid, present a peculiar baccated appearance. The size and relative position of their portions or divisions differ in various series of animals. In the human subject each compartment is of an oval shape, and its long small extremity is in apposition to the one next adjoining. In some species of the monkey-tribe, the fibre appears to be composed of two rows of compartments parallel to each other. In the ourang-utan the fibre is composed of rhomboidal divisions. In another part of the work he states that the convoluted fibre of the pulp appears, on microscopic observation, to be made up of single successive granules. Nothing can show more forcibly the progress made by the author in this department of science, and the extent to which he differs from previous enquiries, than the fact that the best writers hitherto, including even Purkinje, Müller, and Retzius, either positively state that the interfibrous substance of the ivory is structureless (*strukturlos* is the word made use of by Müller in speaking of Purkinje's researches), or else, though it forms four fifths of the substance of the tooth, pass it over without notice. Mr. Nasmyth appears to have proved the fact of its organization both synthetically and analytically; and from a microscopic examination of perfect ivory, he concludes that it presents in different animals peculiarities so remarkably well defined as to furnish a most important and interesting accession to the odontographic basis for a classification of the animal kingdom. Nothing can be more distinct from the interfibrous cells described by Mr. Nasmyth than the corpuscles or cells, represented by Retzius as existing, though scarcely demonstrable, in human teeth, towards the periphery of the ivory, and in which, according to him, the dental tubes terminate.

The enamel, from Mr. Nasmyth's observations, appears to present compartments or divisions, but of a different character from those of the interfibrous substance of the ivory. Each compartment of the enamel is of a semicircular form, and the convexity of the semicircle or arch looks upwards towards the free external portion of the tooth. The enamel, the author states, is covered in all instances by a layer of *crusta petrosa*, in which he has distinctly traced the corpuscles of Purkinje analogous to those found in bone.

*The Epithelium.* This Mr. Nasmyth defines to be a layer of non-vascular but yet organized substance, covering the vascular surface of mucous membranes. Both the cuticle and epithelium are formed, it appears to him, from a fluid secretion on the surface of the chorion. They are composed of particles which pass through various stages of development, viz., first, that of the formation of nuclei and corpuscles; second, that of cells; third, the growth of the latter by vital imbibition; fourth, their compression and gradual conversion into minute lamellæ or scales. In a section of the epithelium and mucous membrane, numerous nuclei are found in opposition to the latter, which more externally are surrounded by a cell, whilst at the surface this cell is found compressed, and assuming the appearance of a scale. The epithelial cells are connected together by an elastic gelatinous substance interspersed with minute granular bodies. From the surface of the skin and mucous membranes, the external layer of scales is continually being thrown off, in consequence of pressure from those in process of development beneath. After cuticular lamellæ have been detached, their place is regularly occupied again by newly-formed scales.

It appears to the author that the component parts of the cuticle and epithelium have within themselves a power of growth; it remains for pathologists to determine what share the derangement of this function has in the production of cutaneous diseases. Mr. Nasmyth concludes this division of his subject by a more detailed description of the epithelium of the mouth, which in the fœtal subject forms a white, dense, projecting layer on the alveolar arch, which has been erroneously termed dental cartilage. In the interior of this alveolar epithelium, where it corresponds to the molar teeth, small vesicles may frequently be observed, varying in size from a quarter to an eighth of a line in diameter. Under the microscope their parietes are found to consist of alternated scales, and their cavity to contain a fluid abounding in minute granules or cells. The internal surface of the alveolar epithelium frequently presents concavities corresponding to larger vesicles on the mucous membrane, which contain floating globules and scales similar to those of the epithelium. Its external surface presents, after slight maceration, numerous fringed processes composed of elongated scales.

---

